# Supplementary material for: Automated vision-based assistance tools in bronchoscopy: stenosis severity estimation
Source: Int J Comput Assist Radiol Surg. 2025 May 15;20(8):1733–40. doi: 10.1007/s11548-025-03398-x (PMC12350464; doi:10.1007/s11548-025-03398-x)
Supplement: Supplementary file 1 — (pdf 185 KB) [file 11548_2025_3398_MOESM1_ESM.pdf]

Automated vision-based assistance tools in bronchoscopy: stenosis severity estimation

Online Resource 1

Clara Tomasini[1], Javier Rodriguez-Puigvert[1], Dinora Polanco[2], Manuel Viñuales[2], Luis Riazuelo[1], Ana C. Murillo[1]

[1]DIIS, i3A. Universidad de Zaragoza, Zaragoza, Spain.
[2]Hospital Universitario Miguel Servet, Zaragoza, Spain.

Contributing authors: ctomasini@unizar.es;

Journal: International Journal of Computer Assisted Radiology and Surgery (IJCARS)

Detailed Per Sequence Quantitative Results

We provide detailed PSA and PSD measurements for each of the 16 sequences of the Subglottic Stenosis Dataset. We calculate Absolute Error between our estimations and ground-truth (GT) PSA measurements and reference PSD estimation respectively. To further evaluate our pipeline, and specifically our proposed segmentation method for keyframe selection, we also compare the PSA and PSD measured at keyframes selected using our intensity-based segmentation, State-of-The-Art segmentation metohds SAM [1] and SLIC [2], and at a manually selected, optimal keyframe. The results are shown in Table 1 for PSA and Table 2 for PSD.

Table 1 Proposed stenosis estimation (Ours) compared to other baselines with different segmentation methods for keyframe selection. PSA Absolute Error (**AE**) computed for 5 sequences from SGS dataset with CT scans.

Seq.	Seg. Method	PSA (%)	GT (%)	AE (%)
1[§]	Ours	58,01	61,51	3,50
1[§]	SAM	No keyframe[‡]	61,51	61,51
1[§]	SLIC	No keyframe[‡]	61,51	61,51
1	Manual	60,87	61,51	0,64
5	Ours	67,57	59,58	7,99
5	SAM	68,83	59,58	8,98
5	SLIC	No keyframe[‡]	59,58	59,58
5	Manual	58,54	59,58	1,04
6	Ours	59,83	62,41	2,58
6	SAM	No keyframe[‡]	62,41	62,41
6	SLIC	78,48	62,41	16,07
6	Manual	61,90	62,41	0,51
7	Ours	62,35	62,41	0,06
7	SAM	No keyframe[‡]	62,41	62,41
7	SLIC	No keyframe[‡]	62,41	62,41
7	Manual	62,35	62,41	0,06
8	Ours	52,35	62,41	10,06
8	SAM	58,54	62,41	3,87
8	SLIC	No keyframe[‡]	62,41	62,41
8	Manual	58,62	62,41	3,79

[§] Sequence used for training the 3D reconstruction model LightDepth only.
[‡] The keyframe is either not detected or detected outside of the correct interval, and the estimation is considered 0.

Table 2: Proposed stenosis estimation (Ours) compared to other baselines with different segmentation methods for keyframe selection. PSD Absolute Error (**AE**) computed for 12 sequences from SGS dataset with expert estimations.

Seq.	Seg. Method	PSD (%)	Ref. (%)	AE (%)
1[§]	Ours	53,95	60	6,05
1[§]	SAM	No keyframe[‡]	60	60
1[§]	SLIC	No keyframe[‡]	60	60
1[§]	Manual	62,86	60	2,86
2	Ours	56,99	60	3,01
2	SAM	No keyframe[‡]	60	60
2	SLIC	58,30	60	1,70
2	Manual	55,70	60	4,30

[§] Sequence used for training the 3D reconstruction model LightDepth only.
[‡] The keyframe is either not detected or detected outside of the correct interval, and the estimation is considered 0.

**Seq.**	**Seg. Method**	**PSD (%)**	**Ref. (%)**	**AE (%)**
3	Ours	62,42	55	7,42
3	SAM	59,37	55	4,37
3	SLIC	No keyframe‡	55	55
3	Manual	62,94	55	7,94
6	Ours	65,85	70	4,15
6	SAM	No keyframe‡	70	70
6	SLIC	62,33	70	7,67
6	Manual	59,09	70	10,91
7	Ours	57,89	60	2,11
7	SAM	No keyframe‡	60	60
7	SLIC	No keyframe‡	60	60
7	Manual	57,89	60	2,11
9	Ours	57,14	65	7,86
9	SAM	74,16	65	9,16
9	SLIC	59,73	65	5,27
9	Manual	65,71	65	0,71
11	Ours	58,13	60	1,87
11	SAM	No keyframe‡	60	60
11	SLIC	No keyframe‡	60	60
11	Manual	58,13	60	1,87
12	Ours	13,33	0	13,33
12	SAM	No keyframe‡	0	0
12	SLIC	No keyframe‡	0	0
12	Manual	5,39	0	5,39
13	Ours	14,86	0	14,86
13	SAM	42,61	0	42,61
13	SLIC	No keyframe‡	0	0
13	Manual	5,56	0	5,56
14	Ours	6,87	0	6,87
14	SAM	10,30	0	10,30
14	SLIC	6,67	0	6,67
14	Manual	10,05	0	10,05
15	Ours	61,21	55	6,21
15	SAM	44,79	55	10,21
15	SLIC	No keyframe‡	55	55
15	Manual	60,43	55	5,43

§ Sequence used for training the 3D reconstruction model LightDepth only.

‡ The keyframe is either not detected or detected outside of the correct interval, and the estimation is considered 0.

Seq.	Seg. Method	PSD (%)	Ref. (%)	AE (%)
16	Ours	36,59	30	6,59
16	SAM	No keyframe[‡]	30	30
16	SLIC	No keyframe[‡]	30	30
16	Manual	36,59	30	6,59

[§] Sequence used for training the 3D reconstruction model LightDepth only.

[‡] The keyframe is either not detected or detected outside of the correct interval, and the estimation is considered 0.

References

[1] Kirillov, A., Mintun, E., Ravi, N., Mao, H., Rolland, C., Gustafson, L., Xiao, T., Whitehead, S., Berg, A.C., Lo, W.-Y., Dollár, P., Girshick, R.: Segment anything. IEEE/CVF Int. Conf. on Computer Vision, 4015–4026 (2023)

[2] Achanta, R., Shaji, A., Smith, K., Lucchi, A., Fua, P., Süsstrunk, S.: Slic superpixels. EPFL Technical Report 149300 (June 2010)
